# Gummatous cutaneous tuberculosis associated with the use of infliximab for Crohn's disease^☆^^☆☆^

**DOI:** 10.1016/j.abd.2020.07.008

**Published:** 2021-01-31

**Authors:** Lucas Campos Garcia, Everton Carlos Siviero do Vale, Maria de Lourdes Ferrari, Lauro Damasceno de Carvalho Faria

**Affiliations:** a Dermatology Service, Hospital das Clínicas, Universidade Federal de Minas Gerais, Belo Horizonte, MG, Brazil; b Department of Gastroenterology, Faculty of Medicine, Universidade Federal de Minas Gerais, Belo Horizonte, MG, Brazil

**Keywords:** Crohn's disease, Tumor Necrosis Factor Receptor-Associated Peptides and Proteins, Molecular target therapy, Skin tuberculosis

## Abstract

As the treatment of infectious and parasitic diseases improved, the prevalence of these conditions declined. However, with the expansion of the use of immunobiologicals, opportunistic infections have emerged, especially under atypical presentations. The present study reports the case of a patient treated with infliximab for Crohn's disease, who presented diarrhea, weight loss, abdominal pain, fever, and subcutaneous erythematous nodules that evolved with spontaneous fluctuation and ulceration. With the finding of alcohol-resistant bacilli and *Mycobacterium tuberculosis* DNA in a cutaneous fragment, through polymerase chain reaction, the diagnosis of gummatous tuberculosis was confirmed, probably secondary to hematogenous dissemination from an intestinal focus.

Immunobiologicals were introduced in the 1990s to treat inflammatory bowel diseases.^1^, ^2^, ^3^ Reactivation of latent infections resulting from the immunosuppression caused by these drugs may occur. Tuberculosis (TB) is more specifically associated with the use of tumor necrosis factor alpha antagonists.^3^

A 26-year-old female patient was diagnosed with Crohn's disease (CD) due to stomach enterorrhagia and abdominal colic. After therapeutic failure with prednisone, mesalazine, and azathioprine, infliximab 5 mg/kg was initiated. Despite the initial improvement in symptoms, she gradually worsened. After 14 months on infliximab, she maintained an active disease and weight loss, in addition to daily feverish peaks. She presented three subcutaneous erythematous nodules on the posterior region of the right thigh, of approximately 3 cm in diameter, without fluctuation or drainage. Subsequently, these lesions ulcerated, with well-defined edges and a necrotic background (Fig. 1). Histopathology showed a nonspecific inflammatory process. Intestinal endoscopic biopsy (Fig. 2) demonstrated granulomas with non-caseous central necrosis and absence of acid-alcohol-fast bacilli (AAFB; Fig. 3).

**Figure 1:**
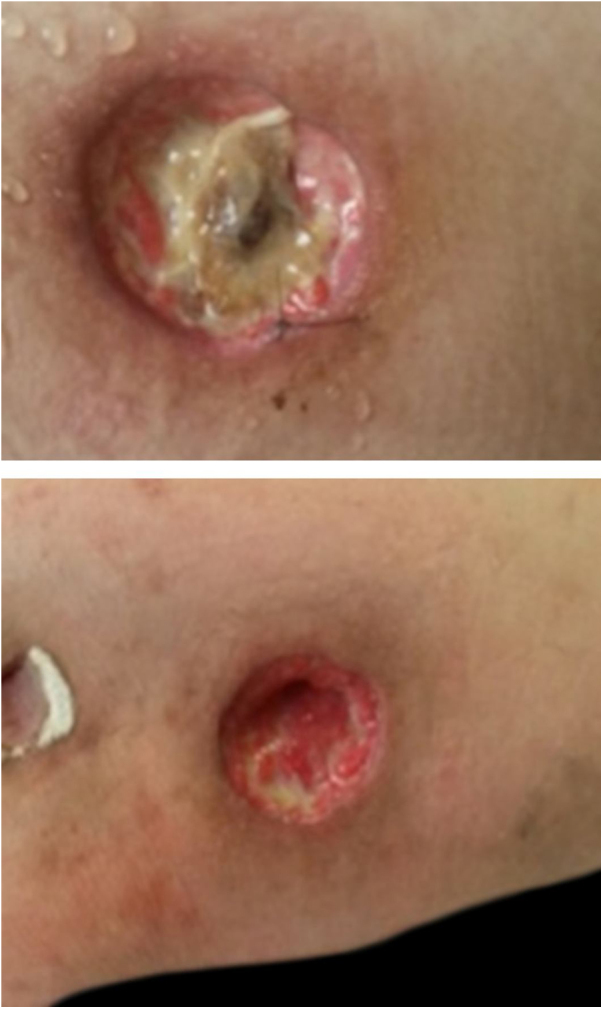
Ulcerated nodules on the posterior region of the right thigh.

**Figure 2:**
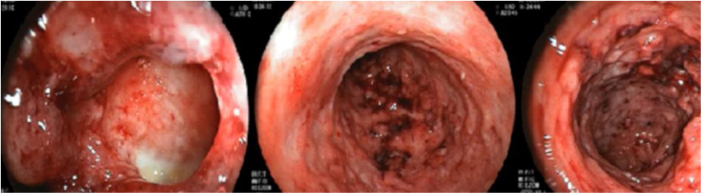
Colonoscopy showing an extensive ulcerated inflammatory process in the colon and ileum.

**Figure 3:**
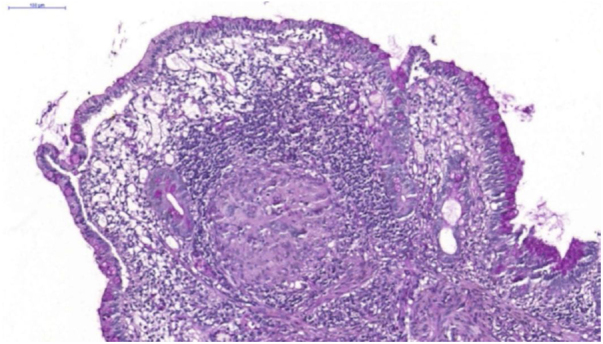
Non-caseating granulomas in the ileal mucosa (Hematoxylin & eosin, ×10).

Due to the characteristics of the granulomas, the hypothesis of infection with *Yersinia pseudotuberculosis* was raised. The treatment with gentamicin and ceftriaxone was ineffective, with the onset of a new erythematous cutaneous nodule on the left thigh, which revealed the presence of AAFB on histopathological examination (Fig. 4) and genetic material of *Mycobacterium tuberculosis* by the rapid molecular test, confirming the diagnosis of gummatous tuberculosis. Computed tomography of the chest showed nonspecific nodules, and pulmonary involvement was not ruled out. Treatment with rifampicin, isoniazid, pyrazinamide, and ethambutol was instituted for six months. The ulcerated nodules healed completely after the third month, with no recurrence after one year of follow-up.

**Figure 4:**
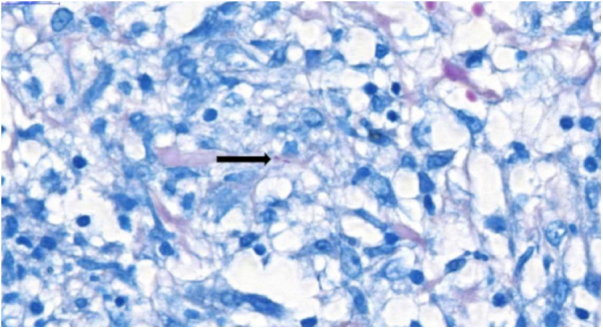
Presence of acid-alcohol-fast bacilli (AAFB; arrow) at histopathological examination of the skin (Ziehl-Neelsen, ×100).

Cutaneous TB accounts for approximately 1% of the cases of the disease.^4^ It can be subdivided into forms caused by direct inoculation, such as tuberculous chancre and TB verrucosa cutis, forms caused by contiguity and autoinoculation, such as scrofuloderma and periorificial TB, and forms caused by hematogenous dissemination, such as gummatous tuberculosis and acute miliary TB. Lupus vulgaris is likely to be caused through the three pathways. Tuberculids are forms in which an exacerbated immune response causes skin lesions, such as erythema induratum of Bazin, lichen scrofulosorum lichen, and papulonecrotic tuberculid.^4^, ^5^, ^6^

Gummatous tuberculosis is rare and affects mainly immunosuppressed and malnourished children. It is characterized by subcutaneous erythematous nodules, which evolve with intense central necrosis, fistulization, and ulceration. It results from the acute hematogenous spread of a primary outbreak during periods of bacillemia. The tuberculin test is usually negative. Histopathology shows extensive necrosis, formation of loose granulomas, and high bacillary load. Molecular techniques also contribute to the diagnosis.^7^ The differential diagnosis includes panniculitis, syphilitic gumma, and hidradenitis suppurativa. The morphology and location of gummatous tuberculosis helps to differentiate it from other multibacillary forms, such as periorificial. The same treatment used for lung disease is recommended. Directly monitored treatment is recommended by the National Tuberculosis Control Program of the Ministry of Health of Brazil, and must be performed at home by primary care.^8^, ^9^ After TB treatment was completed, a good response to ustekinumab for Crohn’s disease was observed.

## Financial support

None declared.

## Authors’ contributions

Lucas Campos Garcia: Approval of the final version of the manuscript; drafting and editing of the manuscript; collection, analysis, and interpretation of data; intellectual participation in propaedeutic and/or therapeutic conduct of the studied cases; critical review of the literature.

Everton Carlos Siviero do Vale: Approval of the final version of the manuscript; drafting and editing of the manuscript; intellectual participation in propaedeutic and/or therapeutic conduct of the studied cases; critical review of the literature; critical review of the manuscript.

Maria de Lourdes Ferrari: Approval of the final version of the manuscript; intellectual participation in propaedeutic and/or therapeutic conduct of the studied cases.

Lauro Damasceno de Carvalho Faria: Approval of the final version of the manuscript; intellectual participation in propaedeutic and/or therapeutic conduct of the studied cases.

## Conflicts of interest

None declared.
